# Sequencing and analysis of the complete mitochondrial genome of *Bactrocera cheni* from China and its phylogenetic analysis

**DOI:** 10.1080/23802359.2020.1715882

**Published:** 2020-01-22

**Authors:** Tao Wang, Yan-ling Ren, Yong Zhong, Mao-fa Yang

**Affiliations:** a Institute of Entomology, Guizhou University, Guiyang, P.R. China;; b Guizhou Light Industry Technical College, Guiyang, P. R. China;; c State key laboratory of tropical and subtropical fruit quarantine, Pingxiang Customs, Pingxiang, P. R. China

**Keywords:** Mitogenome, Dacinae, *Bactrocera cheni*, phylogeny

## Abstract

The complete mitochondrial genome (mitogenome) of the *Bactrocera cheni* (Diptera: Tephritidae: Dacinae) are sequenced and annotated. The mitochondrial genome is 15,945 bp (GenBank No. MN883026), with A + T% for the whole sequence = 73.0% (38.9% A, 16.4% C, 10.6% G, and 34.1% T), which is the classical structure for insect mitogenome. All PCGs started with ATN except ATP8; 9 PCGs use TAA as the stop codon, and others use TAG as the stop codon. The phylogenetic tree confirms that *B. cheni* and *B. tsuneonis* are not clade into one branch with strongly supported. And Pairwise Identity is 80.0% between *B. cheni* and *B. tsuneonis*. Based this study, we supported that *B. cheni* and *B. tsuneonis* are two different species clearly.

Orange fly is considred to be one of the most serious pests damaging oranges and grapefruit, which is distributed in Japan, China and Vietnam, due to its prevention and control worldwide, people should pay substantial attention to them (Wang 1996). *Bactrocera cheni* was named and described by Zhao from a male holotype, plus 33 male and 25 female paratypes, from Guangxi, Hunan, Jiangsu and Sichuan (Zhao 1987). White and Wang had studied types of *B. cheni* from Guangxi and found them to be identical to *Bactrocera tsuneonis* (White and Wang, 1992), and it has been recognized by most studies. But according to *16S* rRNA and *COI-COII* gene sequences, Li et al believed that *B. cheni* and *B. tsuneonis* are very closed different species (Li et al. 2009). In this study, we sequenced and determined the complete mitochondrial genome (mitogenome) of *B. cheni*.

Total genome DNA was extracted from a male adult of *B. cheni* which was collected in Chongzuo City, Guangxi Zhuang Autonomous Region, China (E 106°50′49″, N 22°3′37″), in May 2019. The genome DNA and specimen are deposited in Specimen storage room of Guizhou Light Industry Technical College, label number of them is GLI-IDT-00130. Mitogenome sequences were assembled and annotated using Geneious Primer (Kearse et al. 2012), additionally, tRNAs were found by MITOS server (Bernt et al. 2013) and tRNA scan-SE server (Lowe and Chan 2016). The ML (Maximum Likelihood) tree was constructed using the nucleotide of 13 protein-coding genes and 2 RNA genes sequences by IQ-TREE v1.6.3 (Nguyen et al. 2015).

The complete mitogenome of *B. cheni* is 15,945 bp (GenBank No. MN883026), contained a typical set of 37 mitochondrial genes and one control region (951 bp). The mitogenome of *B. cheni* exhibited heavy AT nucleotide bias, with A + T% for the whole sequence = 73.0%. All PCGs started with ATN (ATA/ATG/ATT/ATC), except ATP8 which started with TTG; 9 PCGs use TAA as the stop codon, and others (*ND3, ND5, ND4* and *Cytb*) use TAG as the stop codon.

The phylogenetic relationships of *B. cheni* were reconstructed with IQ-TREE using an ultrafast bootstrap approximation approach with 10,000 replicates based on concatenated the nucleotides of the 13 PCGs and 2 rRNAs with 13,101 bp (Figure 1). Each PCG and rRNA sequence was aligned using the MAFFT algorithm in TranslatorX and MAFFT v7.0 online serve with the G-INS-i strategy respectively, and aligned sequences were eliminated using Gblocks 9.1 b (Abascal et al. 2010; Katoh et al. 2017). Within phylogenetic tree, *B. cheni* as the sister group of *Bactrocera tryoni* with strongly supported (bootstrap support value = 100). *B. cheni* and *B. tsuneonis* are not clade into one branch with strongly supported. And compared two sequences, the pairwise Identity is 80.0% between *B. cheni* and *B. tsuneonis*. Thus, based this study, we supported that *B. cheni* and *B. tsuneonis* are clearly two different species which different from traditional ideas, and we hope that our data can useful for further study.

**Figure 1.**
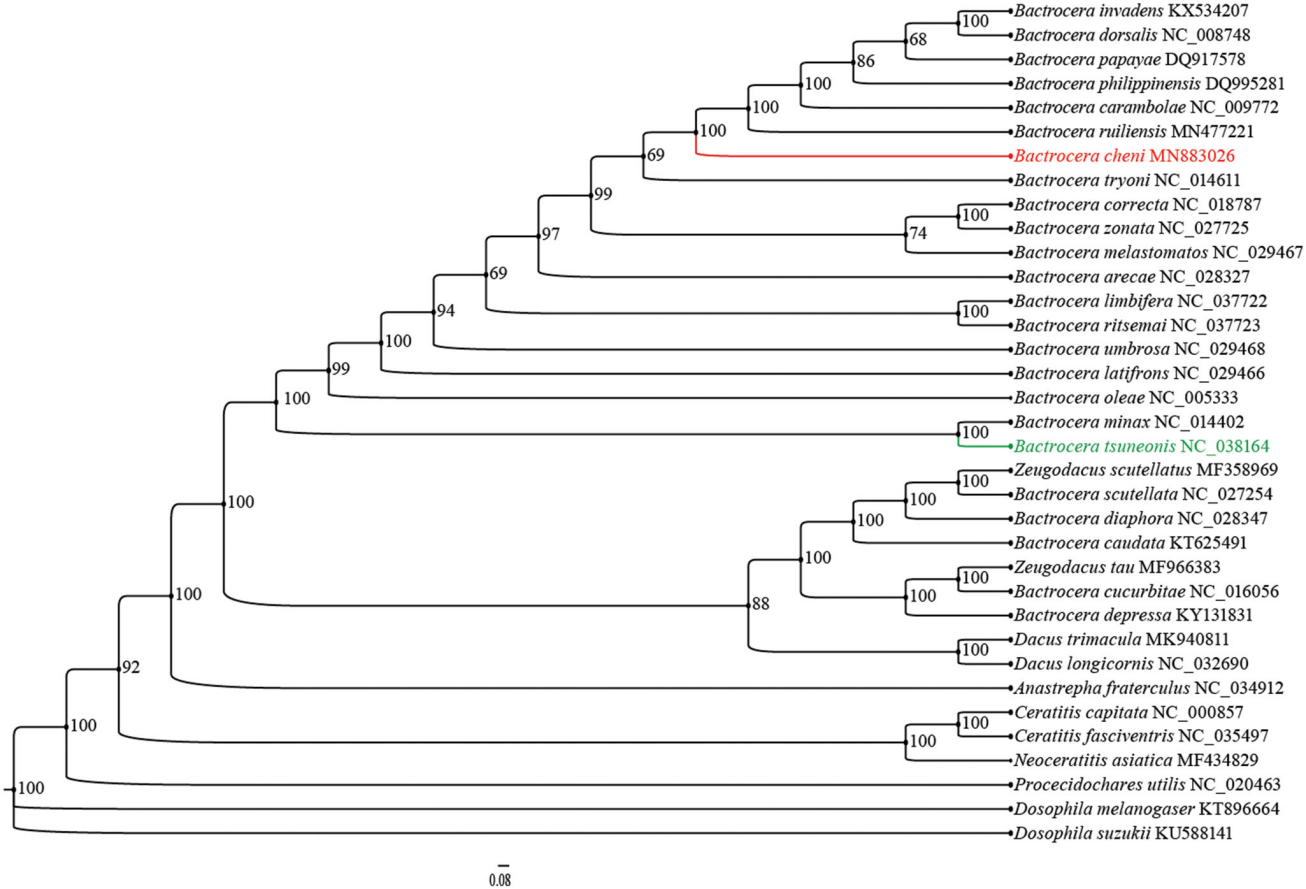
Phylogenetic analyses of *Bactrocera cheni* based upon the concatenated the nucleotides of the 13 PCGs and 2 rRNAs of 32 ingroup species by IQ-TREE. Numbers at nodes are bootstrap values. The accession number for each species is indicated after the scientific name.
